# The Primacy of Skilled Intentionality: on Hutto & Satne’s the Natural Origins of Content

**DOI:** 10.1007/s11406-015-9645-z

**Published:** 2015-11-21

**Authors:** Julian Kiverstein, Erik Rietveld

**Affiliations:** 1 Institute for Logic, Language and Computation (ILLC), University of Amsterdam, Amsterdam, Netherlands; 2 AMC/ILLC/Amsterdam Brain & Cognition, University of Amsterdam, Amsterdam, Netherlands

**Keywords:** Skilled intentionality, Situated normativity, Affordances, Embodied cognition, Higher and lower cognition

## Abstract

Following a brief reconstruction of Hutto & Satne’s paper we focus our critical comments on two issues. First we take up H&S’s claim that a non-representational form of ur-intentionality exists that performs essential work in setting the scene for content-involving forms of intentionality. We will take issue with the characterisation that H&S give of this non-representational form of intentionality. Part of our commentary will therefore be aimed at motivating an alternative account of how there can be intentionality without mental content, which we have called skilled intentionality. Skilled intentionality is the individual’s selective openness and responsiveness to a rich landscape of affordances.

A second issue we take up concerns the distinction between ur-intentionality and content-involving intentionality. We will argue that our notion of skilled intentionality as it is found in humans cuts across these two categories. Instead of distinguishing between different forms of intentionality we recommend focusing on how skilled intentionality takes different forms in different forms of life.

## Introduction

Hutto and Satne’s (H&S’s) paper promises to reignite the debate about how to find a place for intentionality in the natural world. This placement problem was among the central problems that occupied philosophers of mind in the 1990s, when Haugeland’s “Intentionality All-Stars” first appeared (Haugeland 1990). However, in more recent years the problem seems to have lost the sense of urgency it once held for so many naturalistically-minded philosophers. Back in the 1990s many papers would begin with Fodor’s solemn words that the truth of intentional irrealism (the view that there is no intentionality in the natural world) would amount to the “greatest intellectual catastrophe in the history of our species” (Fodor 1987: xii). If H&S are right, the reason interest in the problem waned lies with the failure of the main theoretical contenders. They show how Haugeland’s neo-Cartesian, neo-behaviourist, and neo-pragmatist positions each ran into sizeable obstacles the proponents of these theories failed to find a way around. H&S take the most compelling neo-Cartesian theory to be those that appeal to a combination of natural selection and learning to explain how mental states acquire content. They argue that neo-Cartesianism failed to deliver an explanation of how natural selection and learning deliver semantically evaluable content (H&S, pp. 7–8). Neo-behaviourist and neo-pragmatist theories both ran into circularity problems of different forms. These theories found themselves presupposing that subjects were in possession of mental states with intentional properties in order to get their respective stories started.

H&S suggest a game changing strategy is needed to inject life back into this debate. The result will be a view of the natural origins of content that borrows something from each of the classical naturalist positions Haugeland discussed. The strategy they propose is to distinguish two forms of intentionality. The first is a content-free, non-representational, variety of intentionality which H&S label “ur-intentionality”. The second is the more familiar representational, semantic-content involving variety of intentionality with all of the hallmarks philosophers are taught to associate with intentionality, such as reference and truth conditions. How exactly is this move supposed to help philosophers to get the game of naturalising content moving again?

Let us consider first the problems that H&S identify for neo-behaviourism. Neo-behaviourists account for content-involving psychological attitudes in terms of a capacity to take up the intentional stance in making sense of our own and other’s behaviour. The neo-behaviourist has two sets of problems which H&S think their distinction can help to resolve. The first relates to the intentional system whose behaviour is being interpreted by adopting the intentional stance. (H&S refer to this system as the “intentional patient” (p. 22).) Neo-behaviourism runs the risk of collapsing into a “mere ascriptivism” in which intentionality is solely in the eye of the beholder. H&S think this particular threat can be avoided however once the neo-behaviourist allows that intentional patients have states with ur-intentionality. Ur-intentionality can be cast in the role of setting the scene for the emergence of content-involving forms of intentionality. Whether this move is effective will be a question to which we will return later in our paper.

A second cluster of problems for neo-behaviourism originates with the intentional agent—the person (or animal) that is adopting the intentional stance. Neobehaviourism owes us an account of the concepts of content-involving attitudes which an agent must be in possession of in order to take-up the intentional stance. Furthermore, making sense of behaviour by reference to content-bearing psychological attitudes requires one to be able to reason about psychological attitudes. Such reasoning seems to require an agent to be able to form psychological attitudes about the intentional states of others. H&S argue that the solution to these problems lies in part with neo-pragmatist accounts of intentionality. They point to socio-cultural linguistic practices as the source of human concepts of content-involving attitudes such as beliefs and desires. The explanation of how we acquire such concepts is to be found in the language games we play in making sense of our own and other people’s reasons for actions (Hutto 2008).

Neo-pragmatist theories however face their own problems. They seem to rely in particular on a mechanism for social conformism which are taken to explain how rule-governed practices get started. One question that immediately arises is therefore how humans came by this mechanism of conformism in the first place. Once we say that all intentionality has its origins in language-mediated practices, how can we then explain the emergence of a mechanism for social conformism in human evolutionary history? H&S suggest again that the concept of ur-intentionality may help neo-pragmatists to answer this worry. Through evolutionary inheritance and learning an animal is “set up to be set off” by certain features of its environment. In the case of humans (and other mammals that live in social groups) this mechanism is embedded in a social context in which an individual can be “set up by others” to be set off by certain environmental features, and can in turn “set up others”. The range of behavioural responses to the environment available to human agents thereby massively expands to include crucially, capacities for language learning. On its own this doesn’t explain how humans came to live in social contexts in which they can be set up by others to respond to the world in ways that agree with social practice. However the problem at least looks more tractable once the agents we embed in social contexts are agents that have intentional states. We can appeal for instance to theories of social pedagogy (Sterelny 2012; Tomasello 1999; Csibra and Gergeley 2009) to explain how such agents bootstrapped themselves into social practice.

In this paper we focus our critical comments first on H&S’s claim that a non-representational form of ur-intentionality exists that performs essential work in setting the scene for content-involving forms of intentionality. We will take issue with the characterisation that H&S give of this non-representational form of intentionality. Part of our commentary will therefore be aimed at motivating an alternative account of how there can be intentionality without mental content (developed in more detail in Rietveld and Kiverstein 2014 and Bruineberg and Rietveld 2014).

A second issue we take up concerns the distinction between ur-intentionality and content-involving intentionality. We will argue that our notion of *skilled intentionality* (Rietveld et al. 2013; Rietveld and Kiverstein 2014; Bruineberg and Rietveld 2014) as it is found in humans cuts across these two categories. We agree however that there is an important distinction to be marked in this vicinity between forms of skilled intentionality for which questions of truth or correctness arise, and varieties of skilled intentionality where all that matters is the individual’s practical grip on the environment. In the latter case there are still norms at work; the person or animal appreciates whether it has an adequate or inadequate grip on the environment. However the norms which the individual appreciates are different from normative standards of correctness or truth. They relate instead to the individual’s grip on the environment, and the extent to which this grip can be improved. We suggest that the distinction H&S make between two forms of intentionality may not be the best way to understand this difference. The source of this difference may instead lie in what we call following Wittgenstein, the “form of life” of different animals. The human form of life is one in which questions of truth and correctness matter, and this is not the case for other animals. Instead of distinguishing between different forms of intentionality we focus on how skilled intentionality applies to the multiplicity of activities found within the human form of life. For some of these normatively regulated activities (e.g., construction in architecture, science, the law) what matters to the individual is getting things right. As we see it, the distinction between content-free and content-involving forms of intentionality is better seen as relating to the activities of humans in the context of socio*material* practices (Mol 2002; Orlikowski 2007). This variety of activities can all be made sense of using the Skilled Intentionality Framework (Fig. 1 in Bruineberg and Rietveld 2014: 4). The distinction between content-free and content-involving intentionality is one that can be accommodated within this over-arching framework.

### Intentionality Without Mental Content

Philosophers playing the game of naturalising intentionality have tended to move all too quickly from Brentano’s idea of intentionality as a mark of the mental to understanding intentionality as a defining feature of mental representation. There may seem to be good reasons for making such a move. Intentionality is the term philosophers have inherited from the Scholastics to refer to the mind’s meaningful directedness at the world. The human mind can be directed at objects that do not exist; humans think about fictitious objects, or about objects that might have existed but do not exist. Moreover, humans think about such non-existent objects in an open-ended number of different ways. Think of the rich variety of stories humans have told. Humans can produce such thoughts, one might naturally suppose because of the mind’s representational powers. It is undeniable that people do engage in representational styles of thinking with all of the philosophically puzzling features just mentioned. However this point shouldn’t lead philosophers to run together, and conflate intentionality and mental representation. Representational styles of thinking we will argue derive from particular types of *skills* acquired in socio-cultural practices. It falls squarely within a category of intentionality we have called skilled intentionality. It is a mistake to conflate intentionality with mental representation because representational thinking is a special instance of skilled intentionality which can also take a non-representational form.

*Skilled intentionality is the individual’s selective openness and responsiveness to affordances*—the possibilities for action the environment offers to animals in a form of life because of the skills and abilities available within this form of life. More succinctly: “affordances are relations between aspects of a material environment and abilities available in a form of life” (Rietveld and Kiverstein 2014: 335). In previous work we’ve developed a radical embodied account (Chemero 2009) of skilled intentionality, analysing this concept as it applies to research in ecological psychology (Rietveld and Kiverstein 2014), emotion psychology (Rietveld 2012), social cognition (Rietveld et al. 2013), and systems neuroscience (Bruineberg and Rietveld 2014; Kiverstein and Miller 2015). In this paper we discuss the concept of skilled intentionality as it applies to the *experience* of whole animals and persons situated in particular behavioural settings.

The most natural place to look for non-representational forms of skilled intentionality is in everyday unreflective action. A person (or animal) acts unreflectively when she actively and skilfully engages in some activity without the guidance of conscious thinking, and without first engaging in an explicit mental act of considering her reasons before she acts. In skilled unreflective action a person or animal is presented with aspects of a situation that are always already marked out in terms of their significance for action. The environment shows up in experience as offering a range of more or less inviting affordances. Crucially however the affordances the environment offers are only accessible to individual agents with the necessary skills. Consider the experience of teaching a child to ride a bike. At first the girl has no access to possibility for self-movement the bike offers. She can ride only with the assistance of an adult running alongside, helping her to find her balance. Gradually however, and through repeated practice the child finds she is less and less in need of the adult’s help. She begins to find her own balance until all of a sudden she can ride without the support of the adult. Before she knew how to cycle herself, no doubt the child understood and knew very well *that* bicycles afford riding. She had seen other people ride them, and understood at least something of what these people were doing. Once she learned how to cycle herself however, the girl experiences the affordances her bicycle offers quite differently. The pedals, the handlebars, the saddle etc. all now offer possibilities she herself is able to act on. She is able to pick up on and access the affordances of her bicycle in a way she couldn’t previously to learning how to cycle. Skill thus changes the nature of the agent’s grasp or understanding of an affordance. It gives the agent “access” (Noë 2012) to, and thus opens her to, possibilities for action that would otherwise be closed to her.

When the girl exercises her newly learned skill she doesn’t (and indeed shouldn’t) think to herself about each of the movements she is making, for example the posture of her body as she turns a corner. Were she to think over each of the many fine, micro-adjustments of her body she makes as she steers her bike, she would pretty quickly lose her grip on the bicycle and fall. She has learned through practice how to act naturally and fluently without giving any of these matters any thought. As she exercises her skills she finds that her movements are drawn from her by the bicycle and by the constantly changing particularities of the surroundings she is moving through. There is no need for her to consciously think about what she is doing to stay seated on the bike. The bicycle in its particular setting calls for certain movements from the girl and she exercises her skill by immediately doing what her continuously changing situation requires of her.

Skilled intentionality of the type we find in unreflective action takes the form of a selective openness to the world. The world we experience when acting unreflectively is always already organised in terms of possibilities for action that matter to varying degrees to the individual. An individual animal or person makes contact with this world not by means of perceptual representations with a mind-to-world direction of fit. They achieve access to the surrounding environment through skilled engagement with the concrete affordances the environment offers them because of their skill. It is skill that gives an individual access to, and opens them to the world (Noë 2012). Nor does the experience of acting require an agent to represent the bodily movements he performs, and the sensory consequences he expects to be brought about through his bodily movements. When an agent exercises skill, repeated practice has brought the agent to the point that they are exquisitely sensitive to the demands of the situation, and the environment literally draws their bodily movements from them. They immediately sense when they are doing something that deviates from what the situation demands of them, and take immediate corrective action in such a way as to gain a better and improved grip on the situation (Merleau-Ponty 1945; Dreyfus 2002, 2014; Rietveld 2008a, b).

On the standard representational view of skills, actions have *world-to-mind* direction of fit, and the experience of acting has a *mind-to-world* direction of causation (Searle 1983). An intention or goal is fulfilled when the bodily movements the agent is making (and their associated sensory effects on the world) fit with those the agent has the intention (or goal) to bring about. An experience of acting has a mind-to-world direction of causation because the agent in acting intentionally has an experience of her bodily movements as being caused by her intention and goals. However, in unreflective skilled action the agent has no intention or goal in mind which she acts to bring about. She therefore has no state of mind of the character of an intention or goal with a world-to-mind direction of fit. Moreover, her *experience* of acting has a world-to-mind (and not a mind-to-world) direction of causation. In acting skilfully she experiences the relevant affordances of her environment as drawing her actions from her (Dreyfus 2002, 2014). She experiences herself as “being moved” so as to continuously improve her practical engagement with the concrete, material affordances of her environment (Rietveld 2008a).

A philosophical interpretation of the person’s relation to the world as mediated by content-bearing mental representations is unfortunate because it introduces a subject-object divide into experience were previously there was none. Such an interpretation posits a subject on the one side of this divide that has a psychological attitude of some type towards a proposition (or mental content construed in some other way). On the other side of the divide, an object is introduced with determinate properties to which the subject stands in a relation of reference. This subject-object interpretation of the mind-world relation is a distortion of the relationship people have with the world as they live their daily lives. Consider for instance, how you relate to the café you frequent regularly to buy coffee. The place itself, the people you meet there, the routines you go through of queuing and ordering all unfold in similar ways each time you visit. There is a regular, reliable rhythm and pattern to what happens when you visit a familiar place like your local café that becomes deeply interwoven into your habitual daily routine. You come to take for granted the regularities and patterned ways of doing things you encounter in such a place until you cease to notice them and stop giving them any thought. Dewey once wrote that the “organism is in nature” not as “marbles are in a box but as events are in history, in a moving, growing never finished process.” (Dewey 1958: 295) Dewey points out that the organism is interwoven with nature; the two combine to form a single dynamic process. The same is true of human beings and the familiar places in which so much of our everyday life plays out. We fail to do justice to this interweaving of human life with the places we inhabit so long as we interpret the mind-world relation in representational, subject-object terms.

Notice that Dewey talks of organisms in general as being interwoven with the natural environments they inhabit, not only of humans. We agree with Dewey that skilled intentionality is not the unique possession of humans, but is writ large throughout the animal kingdom. Non-human animals are also selectively responsive to the possibilities for action the environment offers. For a simple example consider Von Uexkull’s description of the life of the tick. Von Uexkull provides a richly evocative description of the woodland environment of the tick, and how from this environment only select aspects “stand out like beacons”, moving the otherwise motionless tick to action (1934: 11). Ticks are sensitive to select aspects of their surroundings. They hang from the branches of trees, and there they remain until they pick up the scent of butyric acid found on the skin of mammals. On encountering this scent, the tick releases its grip so as to fall onto the body of the passing mammal below. Once it makes tactile contact with the animal’s fur it then runs about until it detects the heat of the mammal’s skin. The detection of heat in turn leads to a boring and burrowing movement. The environment of the tick shrinks to just these aspects of smell, touch and warmth, and each of these aspects has an immediate implication for how the tick acts. It is just these aspects of the environment that affect the tick immediately moving it to action. Each possibility however relates in some way to an aspect of the tick’s lifestyle and its related sensitivities. The aspects of the environment to which the tick is selectively sensitive are aspects that mean something to the tick in the context of its comparatively simple way of life.

Indeed we would go further and claim in general that the niche of any kind of animal is composed of those aspects of the environment to which the animal is sensitive because of its form of life. We define the form of life of a kind of animal as the patterns in its activities, its “relatively stable and regular ways of doing things” (Rietveld and Kiverstein 2014: 328). These patterns in the behaviour of a kind of animal are a reflection of the animal’s needs and concerns. At the most basic biological level they relate to the animal’s drive to maintain itself, and resist disorder in its interactions and energetic exchanges with the environment. However animals don’t only care about survival, their own welfare and that of those close to them, and humans in particular have many other interests or concerns. Humans have a rich variety of coordinated ways of interacting with each other that have solidified and become entrenched in social practices and customs. The patterns that stabilise in these coordinated ways of interacting are a reflection of the projects that matter to us humans. If we want to understand skilled intentionality in naturalistic terms, we must take into account the form of life of an animal. This means also having in view what the animal cares about, or the meaning it gives to the environment. The patterns of behaviour that stabilise over the course of an animal’s development are a reflection of that animal’s concerns; of what matters to it. If we ignore the concept of a form of life, we will miss what makes the environment significant for an animal. However it is precisely this significance that we ought to be interested in explaining when we look for a naturalistic explanation of intentionality.

Our aim in this section has been to outline some of our motivations for developing a non-representational account of intentionality. No doubt the reflections we have been offering in this section will fail to persuade philosophers already committed to providing a representational theory of intentionality. H&S have however argued that any naturalistic account of intentionality needs to make room for a non-representational species of intentionality, and we fully agree. We are proposing skilled intentionality as the best candidate for occupying such a position in the theoretical space they have mapped out. Our notion of a skilled intentionality, understood as the individual’s selective openness and responsiveness to a rich landscape of affordances, captures best the relationship that humans and non-human animals have with the environment as they live out their everyday lives. In the next two sections we turn our attention to H&S’s concept of ur-intentionality. We suggest some necessary refinements to their concept that follow from the arguments we have given so far.

### From ur-Intentionality to Skilled Intentionality

H&S motivate the need for a non-representational account of intentionality in a very different way from us. They point to the problems that have arisen for naturalistic accounts of intentionality that attempt to ground intentionality in an organism’s evolutionary history. These attempts fail to explain how it is that mental representations can be directed towards specific objects under a particular aspect.

H&S suggest however that selectionist-style explanations fare much better when it comes to explaining “why certain organisms are responsive to a selective range of worldly items” (H&S, p.21). The frog has been set up over the course of its evolutionary history to have snapping type behavioural responses set off by flies (but also by other small black moving objects that resemble flies). They then equate ur-intentionality with “response tendencies” that because of an animal’s evolutionary history are targeted at particular environmental targets. The advantage of making this move is that selectionist-style explanation is now given the task of explaining why an organism has what we will call “targeted response tendencies”. It doesn’t matter that targeted response tendencies (henceforth abbreviated as “TRTs”) are set-off by an open-ended disjunction of different items, since the aim of a naturalistic theory of intentionality is first of all that of explaining ur-intentionality, or the TRTs of an organism. It should thus cease to be a cause for worry that evolutionary history fails to explain how mental states come to possess properties associated with content such as a truth conditional semantics, aspectual shape, etc. The task of explaining these features will be that much easier once one has an account of ur-intentionality in place. H&S take their work as therapists to be done.

Does the concept of ur-intentionality understood as TRTs pass the test we identified at the end of section 1 for a satisfactory naturalistic account of intentionality for animal minds and humans alike? Does it take into account what we’ve described as the form of life of a species of animal? We argued in the previous section that the notion of a form of life ought to occupy a central place in a naturalistic explanation of intentionality (see also Rietveld and Kiverstein 2014). The significance and meaning an individual animal gives to the environment comes from the form of life to which the animal belongs. It originates in the recurrent and coordinated ways of interacting with the environment found among the members of species. We suggested above that it is a constraint on the adequacy of a naturalistic theory of intentionality that it accommodates this concept of a form of life. Does H&S account of ur-intentionality (or the reconstruction of it we offer below) pass this test?

H&S say very little about how to conceive of TRTs, but what they do say makes this concept sound a lot like what Wheeler has (2005) dubbed “situated special purpose adaptive couplings” in his analysis of research in situated robotics. Consider again the example of the frog that H&S rely on. The frog’s fly-snapping mechanism is a sensorimotor mechanism. If H&S are right about ur-intentionality, this mechanism doesn’t work on the basis of representations stored inside of the frog’s head. Instead it works through the dynamic interactions that unfold between neural components, non-neural bodily components and features of the environmental surroundings. The mechanism is “special purpose” in the sense that it is set-up (i.e., hard-wired) to be triggered only by specific features in the environment. Wheeler explains the idea in the following way in discussing the related example of cricket phonotaxis:

…the cricket’s special purpose mechanism, in the very process of being activated by a specific environmental trigger, brings a context of activity along with it, implicitly realised in the very operating principles which define that mechanism’s successful functioning. (Wheeler 2008: 335)

The fly-snapping mechanism only works (i.e., it only results in the frog catching a fly) when it fires in response to small black moving objects. Built into the mechanism’s operating principles is the context in which the mechanism functions (i.e., a context in which there are black moving objects present). It is only in this context that the mechanism functions at all. This may remind the reader of the example of Von Uexkull’s tick which we discussed earlier. The tick’s restricted repertoire of behaviour can likewise be described as resulting from mechanisms that function by responding exclusively to specific cues. In a context in which the tick is running around on the back of an animal, for instance, the mechanism that is active is one that responds only to the skin of the mammal, and it is only once the skin of the mammal is sensed that the tick commences its burrowing.

The concept of TRTs faces a number of problems. First, it implies a problematic view of animal behaviour as either hard-wired or learned dispositions to respond to fixed and stable environmental cues. The selective responsiveness of animal behaviour which we have taken to be constitutive of skills is thereby conceived of as the animal being equipped with mechanisms that are set off only by specific environmental triggers. We will suggest a different conception of the selective responsiveness of animal behaviour that shows up the inadequacy of the TRT concept. An animal is never selectively responsive to only a single affordance, but to a whole field of affordances each of which has a continuously changing degree of relevance for an individual animal (Rietveld 2012, pp. 122–126). We use this point to dispute H&S’s claim that the non-representational species of intentionality is best understood as ur-intentionality.

Based on ideas drawn from the work of the emotion psychologist Nico Frijda, we argue that an animal is selectively responsive to affordances that matter most to the animal because of its needs and concerns; i.e., because of what it cares about (Frijda 1986, 2007). Many (but probably not all) animals are behaviourally responsive to *multiple* relevant affordances simultaneously or to what we will call a whole “field of affordances” each of which is of greater or lesser significance to the animal. The significance of an affordance manifests in the urgency with which the affordance is currently moving the individual to ready itself for action. For example, when the animal is hungry, food will most likely exert the strongest pull on its behaviour. Suppose now the food is present at a location in the plain view of a predator. Then the animal biding its time in the safety of a hole will most likely be felt more strongly to be the most attractive course of action. Some of the possible actions the environment offers will strike the animal as immediately relevant, and demand that the animal immediately acts on them now. Others exert a less urgent, but nevertheless non-negligible pull on the animal’s bodily behaviour. The animal is still somewhat ready to act on these possibilities, but they don’t matter as much to the animal at the time as other of the possibilities on offer. Still other possibilities go completely ignored because they matter not one bit to the animal at the time.

The individual is thus open to and ready to act on multiple affordances at the same time. Why is this first qualification necessary? Building upon our definition of affordances as relations between aspects of the material environment and abilities available in a form of life, we distinguish the field of affordances from what we call the landscape of affordances:

The landscape of affordances: the possibilities for action available in a particular form of life because of the patterned and coordinated activities in which members of this form of life are *able* to partake in.

The field of affordances: the multiple possibilities for action that stand out as relevant for an individual in a particular situation because of their needs and concerns.

An individual animal is thus selectively responsive to the landscape of affordances at the particular place at which the animal is located. The result of this selective responsiveness is readiness to respond to the multiple affordances that make up the field of affordances. (For more on the importance of states of action readiness see Bruineberg and Rietveld 2014, pp. 2–4, pp. 10–11).

The *experience* of an individual animal which we characterise in terms of skilled intentionality thus has the following two features:

Selective responsiveness to landscape of affordances based on the concerns and needs of the animal.

Openness to a field of affordances understood as different degrees of readiness of the individual animal to act on multiple affordances because of its skills and abilities.

How is it that from the landscape of affordances certain possibilities come to stand out as relevant to an individual, and thus as making up the field of affordances for the individual at this time? This is the question we think must be answered if a naturalistic account of skilled intentionality is to be given. The resulting philosophical theory will span multiple levels of description as we have already briefly mentioned above (see Bruineberg and Rietveld 2014 for such an account). We’ve focused so far on the experience of the individual person or animal belonging to a particular form of life, coping more or less adequately with the environment in its skilled activities. Selective responsiveness and openness to a field of affordances can also be understood at psychological and neural levels. It is at these levels of description that a naturalistic account of skilled intentionality can be framed. The concept we have introduced to understand skilled intentionality at the psychological and neural levels is the tendency towards an optimal grip on a field of affordances (Bruineberg and Rietveld 2014; Rietveld 2008b).

The grip an animal has on the field of affordances is never optimal. Living systems are always simultaneously “in a state of relative equilibrium and in a state of disequilibrium” (Merleau-Ponty 1968/2003: 149; Rietveld 2008b, ch.7). The organism is in a state of inherent instability in its interactions with the environment, constantly having needs it must satisfy in order to keep itself alive and to function well:

“This disequilibrium or absence inspires or motivates self-organized compensatory activity or ‘auto-regulatory fluctuation’ (Merleau-Ponty 1968/2003: 149). For example, the organism itself repairs its tissue damage, or restores its energy level by searching for food or by sleeping […]” (Rietveld 2008b: 224).

The reduction of disequilibrium is also seen in more complex improvements of a person’s situation in the context of socio-cultural practices, such as an architect changing the size of a door (Rietveld 2008a), or a person making an effort to comfort a friend who just suffered a loss. Importantly:

“Even though what is absent or lacking and generates the perturbations is ‘not a lack of this or that’ (Merleau-Ponty 1968/2003: 155), the active organism can reconquer (relative) equilibrium.” (Rietveld 2008b: 224).

Or to put it differently, living organisms can be understood as being structurally in a state of inherent disequilibrium. Thus its lack or absence can never be filled completely (Hansen 2005: 243–244). “Re-equilibration should therefore be understood as *relative re-equilibration*.” (Rietveld 2008b: 227).

Thus any living creature will tend towards a state of relative equilibrium with the environment. This striving is what makes some affordances stand out as *relevant* affordances. So the core idea behind our interpretation of the tendency towards an optimal grip is that the animal in its interactions with the environment will always be drawn to and attracted by those action possibilities the responsiveness to which will improve its situation. The organism in its dynamical interaction with the environment forms a far from equilibrium, self-organising system. The dynamics of this self-organising process are such that the organism will find itself drawn to affordances that move it closer to equilibrium with the dynamically changing environment (see Bruineberg and Rietveld 2014). Responding to (or being poised to respond to) an action possibility will only move an animal closer to equilibrium when such a response contributes in some way to an improvement of the animal’s overall situation. Reducing hunger is the most obvious example of what we have in mind. The organism has a metabolic need for energy and thus is in a potentially unstable and threatening relationship with the environment. Taking action and finding food restores the organism’s energy needs temporarily, thereby moving the organism (with respects to its energy needs) closer towards relative equilibrium with its environment. The organism will inevitably have other needs and concerns that make it the case that other affordances have to be acted on to reduce the level of dis-attunement with the dynamically changing environment. Within the Skilled Intentionality Framework, it is the tendency towards an optimal grip that explains the individual’s selective openness to *multiple* affordances in a particular situation (Bruineberg and Rietveld 2014). It is not clear to us how H&S’s concept of ur-intentionality could explain this readiness to act on multiple action possibilities that is characteristic of experience from moment to moment. We return to this point below.

The multiple aspects of the landscape of affordances that stand out as relevant affordances are those aspects of the environment that the organism should be ready to act on if it is to improve its grip on the situation. These multiple affordances immediately elicit in the animal a corresponding set of action readiness each of different strengths because of how the animal is fairing in its interactions with the world. The aspects of the landscape that make it into the field of affordances of an individual animal are therefore always those that are of affective significance to the animal. The skilled individual animal and the landscape of affordances together form a coupled self-organising dynamical system. The dynamics of this self-organising system are such that the individual finds itself drawn to those aspects of the landscape of affordances that relate to what the animal cares about.

So far we have contrasted our view of selective responsiveness to a landscape of affordances with H&S’s view of ur-intentionality, and the notion of targeted response tendencies (TRT’s). We haven’t yet said why our view should be preferred. It is worth noting that the two views are compatible. Evolution and learning could indeed equip animals with abilities that manifest themselves in response tendencies that are triggered only by very specific features of the environment. The tick might be an example of this. However, we believe our account of skilled intentionality has primacy over H&M’s concept of ur-intentionality for the following reasons.

First, the notion of TRTs misses the skill that is involved in responding adequately to the affordances the environment offers. We have seen above how an aspect of the environment offers affordances that an individual can only pick up when the individual has the necessary abilities to engage adequately with the affordance. Without these abilities the individual’s encounter with this affordance provides them with no practical grip or understanding of the possibility the environment offers. Without this practical understanding, the individual has no access to the possible actions the environment furnishes. The individual quite simply wouldn’t understand what opportunities for action the environment was offering. In order for an individual agent to have access to an affordance available in the environment, the individual must know-how to act on that possibility (Noë 2012). Targeted response tendencies are cheap however – missile guidance systems and thermostats have them. They do not require skill or understanding on the part of the mechanism that is responding.

We take the understanding of the environment that comes with skill to be related to what we have earlier called the tendency to optimal grip on a field of affordances. The latter concept can be applied at all levels of description of the animal environment system: the level of the whole animal in its interactions with the environment, but also at the psychological and neural levels. Skill is exercised in acting so as to improve one’s grip on a situation and one’s sense of what the situation demands from one as one acts. The agent in acting skilfully has a sense of how well they understand what the situation is inviting them to do as they act. They experience this depth of understanding as an affective tension. The further the animal is from relative equilibrium in its dealing with the environment, the greater the resulting affective tension. In acting so as to move closer to equilibrium, the skilled agent is also acting so as to reduce this affective or felt tension. Indeed a crucial part of skill is knowing how to act so as to reduce this felt tension with the environment. It is being able to do this that gives one a grip on, and thus an understanding of a possibility for action the environment offers. The individual’s understanding of how it should respond to the landscape of affordances in its ecological niche can always be further improved. This is true even in the most expert of individuals, and this is why even experts never stop learning what they can do with their skill.

A second and more problematic reason we find the H&S characterisation of ur-intentionality incomplete is because it fails to capture how in perception the animal is open to a *whole field* of affordances, which it selects from the landscape of affordances. We’ve seen above how an animal is always ready to respond to more than just the affordance that it is currently acting upon. As the rabbit nibbles on a carrot, a rustle in the nearby bushes can instantly transform the shape of its field of affordances. The carrot ceases to matter so much (but not entirely), and reaching the nearby hole in the ground becomes the most attractive and inviting of the possibilities the environment offers. The hole triggers the strong need to hide when the rabbit senses danger, or when it is tired and needs to find somewhere safe to sleep. The animal’s perception of the environment is always rich with affective significance. The environment offers all manner of attractive opportunities, but also threats to the animal’s well-being that demand action readiness. As the animal responds to a particular invitation to act, *it is simultaneously sensitive to and ready to act on a whole range of other possibilities*. It is the readiness for these other affordances in the field that is crucial for understanding anticipation and how we are able to switch from doing one thing to another without forming a goal or intention (Rietveld 2012). By equating ur-intentionality with response tendencies that are triggered by specific cues, H&S miss this openness to other action possibilities that lie on the horizon for the animal because of its simultaneous readiness to engage with multiple affordances. They also miss the way in which the field of affordances is in constant flux. The degree of relevance assigned to each of the affordances that make up the field of affordances continuously varies according to how the affordance bears on the reduction of the dis-attument/dis-equilibrium that is inherent in the animal-environment system.

### Two Forms of Intentionality?

We end our discussion by raising some concerns about the distinction that H&S make between ur-intentionality as a content-free type of intentionality, and representation-based, content-involving forms of intentionality which they look to neo-behaviourism and neo-pragmatism to explain. We don’t doubt that there is a distinction to be made here between representation-involving and non-representational forms of intentionality. Still important questions remain about how best to mark this distinction. One result H&S clearly would not like to end up with is a neo-Cartesian theory of the content-involving type of intentionality. H&S restrict neo-Cartesian theories to first base theories that apply only to content-free forms of ur-intentionality. Does this mean that they wish to deny altogether that folk psychological attitudes with mental content are to be found inside of the heads of persons and animals? Neo-Cartesianism (as Haugeland originally characterised it) is an industrial-strength realist theory of folk psychological attitudes. In arguing that neo-Cartesian theories only apply to content-free ur-intentionality, do H&S mean to argue that there is no mental content inside of the heads of the subjects whose behaviour we explain in folk psychological terms?

It could be that H&S mean to argue for a slightly less radical position that there is no mental content internal to subjects without social and cultural practices. Animals that do not participate in practices of narrative construction for instance lack content-involving folk psychological attitudes. This move might strike many philosophers working on animal behaviour as a tough bullet to bite in that it would require them to say that animals do not have beliefs and desires, hopes and fears. It would also seem to invite hard questions about how such practices form the basis for anything other than *ascriptions* of content-involving mental content to humans. It is hard to see how ur-intentionality combined with social and cultural practices adds up to internalised mental content. The work of the early twentieth Soviet Psychologists on the role of communicative sign systems such as speech, writing, diagrams, maps and drawings and numerical systems in cognitive development could very possibly help with this last point (Vygotsky 1978; Bruner 1997; Wertsch 2007). Still significant questions remain about how sociocultural products “manage through social interaction to get from ‘outside’ into our ‘inside’ repertory of thought” (Bruner 1997: 66). Doesn’t ‘internalization’ of communicative sign systems require some sort of ‘external-internal’ conversion? Is the result of this process of conversion once again 1internal representational vehicles bearing semantic-content?

Furthermore, what do H&S make of the arguments in Haugeland (1998) for firmly distinguishing what he called the “ersatz intentionality” found in animals and situated robots from the intrinsically normative variety of intentionality found in humans (Haugeland 1998, ch’s 12 & 13)? Haugeland’s category of ersatz intentionality is not unlike H&S’s category of ur-intentionality. It applies to intentional systems that can respond in targeted ways to their environment. Haugeland argued (mistakenly in our view) that the intentional states of robots and animals have a type of intentionality and normativity that is always “conferred from the outside” (Haugeland 1998: 303). His comparison of intentionality in animals and robots with biological teleology is instructive in this regard:

“We say that the “purpose” of the heart is to pump blood, that it’s “supposed” to work in a certain way, that functional descriptions are “normative” and so on. This is not mere as-if teleology… But finally, of course, the heart does not have any purposes in the way that a person does, nor does it accede to any norms on its own responsibility.” (Haugeland 1998: 303)

The key difference between humans and non-human animals for Haugeland can be traced back to normativity. Only human have intrinsic intentionality because only humans have normative standards for what counts as “objects, adequate representations, goals, success and so on” (op cit, p.302). Just as the heart has no purposes of its own on the basis of which it pumps blood, so animals have no normative standards of their own on the basis of which they count representations as true or adequate to objects.

We suspect H&S would not agree with Haugeland that ur-intentionality is ersatz intentionality, an inferior imitation of the real thing, and they would be right not to do so. We will have more to say about this last point in a moment. How can H&S resist Haugeland’s characterisation of ur-intentionality once they distinguish between two forms of intentionality? In discussing the socio-cultural practices and norms that are appealed to by neo-pragmatists, H&S quite rightly describe these practices as “truth-telling practices”. These practices involve representing “things as being thus and so, independently of what we say about them” (p.26). They go on to argue that truth-telling practices “contain a special sense of going wrong”, “a sense of being correct or incorrect according to how things are anyway”. Haugeland takes real, authentic intentionality to be tied exclusively to such truth-telling practices. Any agent or system that doesn’t commit to taking part in such practices lacks authentic intentionality. H&S owe an explanation as to why they don’t share this conclusion. Why do they think that ur-intentionality is not ersatz intentionality?

H&S are certainly right to insist on the existence of truth-telling practices, but this claim would seem potentially to make additional trouble for them. They owe us an explanation of how such practices emerge or evolved out of social conformism and the capacity to adopt the intentional stance. Haugeland strongly doubted that social-cultural norms could account for the difference between what he called “proper performance” and “getting things right” (1998: 313).^1^ Proper performance is a type of rule-following in which the rule decides whether someone has acted properly or improperly. Social conformism determines whether an instance of rule-following behaviour counts as a proper or improper performance. Whether an individual’s action counts as a proper performance or not is certainly constrained by something independent from the individual, but in the end this constraint comes from the community at large. Haugeland claims we cannot make sense of the majority of the community being wrong about whether some case of rule-following counts as a proper performance. Haugeland argues that truth-telling practices are different in this regard. Whether we tell the truth or not in making an assertion is Haugeland claims decided by norms of objective correctness or truth. For Haugeland norms of objective correctness that apply to truth-telling practices are not social norms in which correctness is determined by consensus. H&S seem to agree with this last point when they make a distinction between truth-telling practices and social consensus. Having insisted on such a distinction how can a neo-pragmatist hope to explain content-involving intentionality when the latter is bound up with truth-telling practices?

We believe our concept of skilled intentionality may help to alleviate some (if not all) of the problems we have just identified. We’ve been arguing in this paper that the affordances the environment offers to an animal are relative to its form of life because they are aspects of its ecological niche. The human form of life exhibits greater diversity than that of any other animal, and it does so, we have argued elsewhere, owing to the differences in the embodied skills of humans situated in particular structured surroundings (Rietveld and Kiverstein 2014). Many of the affordances the environment furnishes to humans have their origin in material culture and the coordinated and patterned ways of interacting with other people and with the material environment that stabilise as social practices, or better, as socio*material* practices (Rietveld and Kiverstein 2014, pp. 332–335). Among these patterned practices are representation-based practices that involve the use of systems of material symbols and signs. These symbol systems take the form of maps and diagrams, but also of words and sentences in a public language. Symbolic systems of representation offer humans many stable and regular ways of doing things. They do so because of the repertoire of skills humans acquire during the extended period of development in which children are immersed in a sociomaterial environment overflowing with symbolically mediated systems of communication.

The notion of skilled intentionality we have discussed above takes the sociomaterial nature of our environment seriously and applies both to symbol-mediated and representation-based forms of communication, and to non-representational forms of intentionality alike. The distinction H&S propose between two forms of intentionality is therefore best viewed as a distinction to be made within the category of skilled intentionality. Human beings are always situated in a landscape of affordances that is richly resourceful in terms of the possibilities for action it offers. Many of these possibilities derive from the socio-cultural and material practices local to different communities. For the people that take part in these sociomaterial practices the environment offers all sorts of opportunities that it doesn’t offer to those that are not initiated.

Consider the linguistic and other type of symbolic practice we have pointed to as the source of representation-based styles of thinking. Language use can be understood within the Skilled Intentionality Framework set out above (see Rietveld and Kiverstein 2014, pp. 343–345). Following Wittgenstein, we view language broadly as a kind of toolbox in which words can be used to do different things within sociomaterial practices. It is thus words that offer language users a rich variety of possibilities for action. Words can for instance be used to names things, and names can be used to categorise objects. As language users, we can use words to point out the difference between a nettle leaf and a mint leaf, which can matter to people in the context of tea-making practices. Words can be used to command and instruct people to do what we want them to do, as in the famous opening sections of the *Investigations* in which Wittgenstein discusses the instructions builders give to their assistants to deal with materials aspects of their environment. In conversation we can discuss people and things that are not present in the environment in which the conversation is taking place. We can talk for instance about our friend Christian’s brother even if Christian in fact has no brother. Language offers the potential for us to imaginatively create worlds that don’t exist, both possible and impossible. It provides the possibility for astronomers of the past like Le Verrier to imagine an extra planet in our solar system orbiting between Mercury and the Sun. Each of these examples of language use is an example of an aspect “of engaged human living” (Noë 2009: 103). Language use is bound up with the multiplicity of sociomaterial practices people engage in. As participants in these practices, we become experts in the ways of using language that belong intimately to these practices including, crucially, the material aspects that have shaped the sociomaterial practices. The activities that over time establish these patterned practices are adapted and dynamically calibrated to the way things are in particular situations. The concrete activities individuals engage in as participants in a practice are adapted to the (changing) details of very particular material situations (Rietveld and Kiverstein 2014: 345).

We have described skilled intentionality in part as a selective responsiveness to the landscape of affordances. What is the aspect of the environment we are responsive to when, for instance we use language to talk about things that don’t exist. We can playfully discuss Christian’s brother, but Christian has no brother. How is this possible? The answer to this question lies in the multiple activities humans perform with language. It is these activities that offer the potential to misname individuals, to assign names to individuals that do not exist. These activities are among the patterned practices that make up the human ecological niche because they belong to the human form of life. The patterned activities in which people engage are just as much aspects of the environment as the natural habitats humans populate. An individual speaker can use language in ways that fall in line with the patterned activities of the other users of this language, or he can fail to do so. If he responds correctly this is because he is responding to an affordance the human environment offers, a possibility for action that is in agreement with the patterned practices in which people of his community engage, as well as being answerable to the material reality of the landscape of affordances itself in which the practice is situated. Among the possible actions that symbolic forms of communication offer is thought and talk about a thing that does not exist. In acting in agreement with a sociomaterial practice, the individual thereby makes his actions understandable to others that engage in this practice. In doing so he is manifesting a selective responsiveness to the affordances furnished by the sociomaterial environment humans inhabit. Affordances for language use are just one of the many affordances available in the rich human landscape of affordances.

Whenever a person uses language they manifest a tendency towards an optimal grip on their situation. Consider what happens when we have misunderstood what someone has said to us in a conversation. This misunderstanding becomes apparent as the conversation continues. I might for instance respond to you in a way that doesn’t make sense given what you have said earlier in the conversation. My grip on the conversation and my understanding of you is not quite adequate. This leads to you having an inadequate and partial grip on what I go on to say in the conversation. One of us will have to take measures to repair and correct for the misunderstanding that has developed. Contrast this with what normally happens when conversation flows freely. I succeed in coordinating and adjusting my response to what you have said, and you succeed in doing the same with me. What I say makes you ready to respond appropriately, and when you act on this readiness this makes me ready to respond appropriately and thus to reciprocate. The result is that I understand you well and vice versa, and the conversation flows. Typically, sharing a situation, including a responsiveness to the same place-affordances, contributes to a well-attuned responsiveness to what the other will say as the conversation continues. It allows one to anticipate well where the conversation is going, and to take whatever steps are necessary along the way to avoid misunderstandings (Kiverstein and Rietveld 2012; Bruineberg and Rietveld 2014). Of course there is always the potential for us to improve the mutual understanding we have accomplished so far. This is one reason we might have to continue the conversation further.

Once we have in view the point we’ve been insisting on in this paper that skilled intentionality takes different forms in different forms of life, we believe H&S’s distinction between two forms of intentionality will look rather different. It is a distinction we can make when contrasting the landscape of affordances of humans with that of other animals. When it comes to humans however we should recognise how some of the affordances that people are skilled at dealing with have the defining features associated with propositional forms of normativity such as truth and reference. The sociomaterial practices with these features are the ones that we’ve suggested will be mediated by language use and the use of other systems of material symbols. An account must therefore be given of the skills required to become a member of a linguistically-mediated practice that does not simply reintroduce mental representations inside of the heads of the individual. It is not clear to us that H&S succeed in avoiding this latter consequence. We fair better in this regard. The very same account of skills we have outlined earlier as the tendency towards optimal grip on a field of affordances also applies to sociomaterial practices in which language and other modes of symbolic communication are put to work. These sociomaterial practices it should be noted, will also often include engagement with non-linguistic affordances (remember Wittgenstein’s (1953) example of the builders and the objects they handle). It would be a mistake to artificially separate our sociomaterial ecological niche into a material domain of “the way things are objectively” and a domain of mere “social consensus” or proper performance. The practices that are found in our human form of life are material—they are situated within a landscape of affordances-, but also social, just as we see in the example of Wittgenstein’s builders. The human landscape of affordances is one that is tightly interwoven with both material aspects and social and cultural practices local to different regions of this landscape. The tendency to optimal grip is a result of the self-organising dynamics of a whole agent situated in a sociomaterial landscape of affordances whose aspects occupy a place within human patterned practices. It is the affordances encountered in patterned practices that an individual has a more or less correct and adequate grip on in acting skilfully.

The task of further developing these suggestions on affordances for language use is one we plan to take up in future work. We conclude by showing how the theory of skilled intentionality we have outlined, may be able to avoid some of the other problems we raised above in pitching Haugeland against H&S.

## Conclusion

We registered above our disagreement with Haugeland’s claim that it is only humans that have intrinsic, original or real intentionality. We begin our concluding comments by returning to this point. Haugeland takes normativity to be uniquely human, and we believe he is mistaken to do so. Normative standards are characterised by Haugeland as involving a particular type of commitment, the commitment to getting things right. He argues that humans stand in the right first-person relations of authorship and ownership to their intentional states for them to count as being committed to getting things right. No non-human animal stands in such a relation to their intentional states because no non-human animal submits itself to normative standards in quite the same way as we do. No other animal make itself answerable to standards of correctness as humans do. Haugeland is clearly right to point out that there is a difference, but he fails to notice that there actually is a type of normativity at work in animal behaviour. This leads him to mistakenly set the benchmark for the possession of intrinsic intentionality too high.

The abilities for engaging with affordances adequately or inadequately are to be found throughout the animal world. To borrow an example from the anthropologist Tim Ingold, the weaver-bird is just as well able to explore the possibilities offered by the environment in weaving its elaborate nest as the human is in weaving baskets or weaving wool to make human clothing (Ingold 2011). Both the weaver bird and the human weaver have abilities to engage more or less adequately with the possibilities the material environment offers for making things. The weaver bird can knit together the materials it uses to build its nest adequately or inadequately, well or badly. Part of the weaver bird’s remarkable ability lies in it being sensitive to what can be done with the *materials* it uses for nest-building. When its grip on those materials is less than optimal the bird will adjust its behaviour accordingly so as to improve its grip on the materials it is manipulating. This tendency to act so to reduce relative disequilibrium with the environment is a part of the bird’s selective and skilful responsiveness to the environment. It is moreover characterised by a type of normativity we call *situated normativity* (Rietveld 2008a). The tendency to reduce disequilibrium with the environment is a description of the animal-environment system as a complex dynamical system. However, the same system can also be described at the level of the whole skilled animal’s practical understanding of affordances in context which can be better or worse to different degrees according to the animal’s level of skill or ability.

The normative standards at work in animal behaviour are not conferred on the animal from the outside. These are standards that originate in the animal’s practical understanding of the possibilities for action the material environment offers. Animals in exercising their abilities and skills are no less capable of refining and improving their grip on the environment, and in doing so they display sensitivity to whether their grip on the environment is better or worse. Some way of engaging with the environment can be better or worse relative to the activities in which animals belonging to a particular form of life take part.

What should we make of Haugeland’s distinction between proper performance and getting things right? Our notion of situated normativity might seem to only cover what Haugeland calls “proper performance”. We’ve argued however that skilled intentionality must always be defined as relative to a form of life. The human form of life is one that is heavily mediated by linguistic forms of communication, and questions of getting things right arise for humans with an interest in communicating about a shared sociomaterial world. We have seen in section 1 how skills and abilities give an animal access to the possibilities for action the environment offers. Language gives humans skills for discerning whether we have things right in the context of (changing) sociomaterial practices. The world (in part through the scientific experiments we perform) can tell us we have made a mistake as when Le Verrier posited an extra planet between Mercury and the sun. Language use is answerable to, and constrained by the sociomaterial world humans use language to engage with.

To conclude we’ve identified three broad areas where we believe the Skilled Intentionality Framework we have outlined has advantages over the account of the origins of content H&S propose. First H&S’s concept of ur-intentionality does not address how it is that the individual agent is open to and ready to act on only the *relevant* affordances in a given situation. They fail to explain how what we have called the “field of affordances” emerges from the “landscape of affordances” available in the ecological niche. We have offered an account of this in terms of the tendency towards an optimal grip (see section 2 above, and Bruineberg and Rietveld 2014 for more details). Second, H&S seem to understand ur-intentionality in terms of Targeted Response Tendencies, but in doing so they miss the way in which many animals enjoy multiple simultaneous states of action-readiness that make them responsive to a whole field of relevant affordances. Finally we have argued that the distinction between two forms of intentionality that H&S propose may invite trouble. Some (if not all) of this trouble might be avoided once it is recognise that humans always find themselves situated within a rich landscape of affordances because of the rich variety of sociomaterial practices in which we engage. We strongly resist any separation of the social practices from the sociomaterial landscape of affordances in which these practices are situated. We start explicitly from a socio*material* environment in flux, which allows us to avoid any artificial separation between the “way things are objectively” and a “social consensus” about how to behave that supposedly would be independent of this material reality. Intentionality is always found within a specific form of life. The human form of life is one in which we care deeply about many things, including getting things right in the assertions and judgements we make about the sociomaterial reality we live in.

## Footnotes

1 Haugeland’s distinction between proper performance and truth telling needs careful handling. Such a distinction might be taken to introduce what would in our view be an artificial separation of social practice from the material surroundings in which the practice is situated. We suggest that proper performance isn’t simply a matter of consensus but is also constrained by the possibilities the material environment offers to which the individual agent is responsive. We cannot however enter into this important issue here.

## References

[CR1] Bruineberg, J. & Rietveld, E. (2014). Self-organisation, free energy minimisation and optimal grip on a field of affordances. *Frontiers in Human Neuroscience, 8*, 599. doi:10.3389/fnhum.2014.00599. 10.3389/fnhum.2014.00599 PMC4130179 25161615

[CR2] Bruner J (1997). Celebrating divergence: Piaget & Vygotsky. Human Development.

[CR3] Chemero, A. (2009). *Radical embodied cognitive science*. Cambridge, MA: MIT Press.

[CR4] Csibra G, Gergeley G (2009). Natural pedagogy. Trends in Cognitive Science.

[CR5] Dewey J (1958). Experience and nature.

[CR6] Dreyfus HL (2002). Intelligence without representation: Merleau-Ponty’s critique of mental representation. Phenomenology and the Cognitive Sciences.

[CR7] Dreyfus HL (2014). Skillful coping: essays on the phenomenology of everyday perception and action.

[CR8] Fodor J (1987). Psychosemantics: the problem of meaning in the philosophy of mind.

[CR9] Frijda NH (1986). The emotions.

[CR10] Frijda NH (2007). The laws of emotion.

[CR11] Hansen M, Carman T, Hansen MB (2005). The embryology of the (in)visible. The Cambridge companion to merleau-ponty.

[CR12] Haugeland J (1990). The intentionality all-stars. Philosophical Perspectives.

[CR13] Haugeland J (1998). Having thought. Essays in the metaphysics of mind.

[CR14] Hutto D (2008). Folk-psychological narratives. The socio-cultural basis of understanding reasons.

[CR15] Ingold, T. (2000/2011). *The perception of the environment: essays on livelihood, dwelling and skill.*(London: Routledge.

[CR16] Kiverstein, J. & Miller, M. (2015). The embodied brain: an argument for radical embodied cognition from neuroscience. *Frontiers in Human Neuroscience,* 9.237. doi:10.3389/fnhum.2015.00237. 10.3389/fnhum.2015.00237 PMC4422034 25999836

[CR17] Kiverstein J, Rietveld E (2012). Dealing with context through action-oriented predictive processing. Frontiers in Psychology.

[CR18] Merleau-Ponty, M. (1945). Phénoménologie de la perception. Gallimard: Paris. English edition: Merleau-Ponty, M. (2012) *The phenomenology of perception* (trans: Landes, D.A.) London: Routledge.

[CR19] Merleau-Ponty, M. (1968/2003). *Nature: Course notes from the Collège de France.* Evanston: Northwestern University.

[CR20] Mol A (2002). The body multiple: Ontology in medical practice.

[CR21] Noë A (2009). Out of our heads: Why you are not your brain and other lessons from the biology of consciousness.

[CR22] Noë A (2012). Varieties of presence.

[CR23] Orlikowski WJ (2007). Sociomaterial practices: exploring technology at work. Organization Studies.

[CR24] Rietveld E (2008). Situated normativity: the normative aspect of embodied cognition in unreflective action. Mind.

[CR25] Rietveld, E. (2008b). *Unreflective action: a philosophical contribution to integrative neuroscience.* ILLC Dissertation Series. Institute for Logic, Language and Computation: University of Amsterdam.

[CR26] Rietveld E, Kiverstein J, Wheeler M (2012). Context-switching and responsiveness to real relevance. Heidegger and cognitive science.

[CR27] Rietveld E, Kiverstein J (2014). A rich landscape of affordances. Ecological Psychology.

[CR28] Rietveld E, De Haan S, Denys D (2013). Social affordances in context: what is it that we are bodily responsive to? Commentary on Schilbach et al. The Behavioural and Brain Sciences.

[CR29] Searle JR (1983). Intentionality: an essay in the philosophy of mind.

[CR30] Sterelny K (2012). The evolved apprentice: How evolution made humans unique.

[CR31] Tomasello M (1999). The cultural origins of human cognition.

[CR32] Von Uexkull, J. (1934/1957). A stroll through the worlds of animals and men. In C. Schiller (Ed.), *Instinctive behaviour.* New York: International Universities Press.

[CR33] Vygotsky LS (1978). Mind in society: the development of higher psychological processes.

[CR34] Wertsch JV, Daniels H, Cole M, Wertsch JV (2007). Mediation. The Cambridge companion to Vygotsky.

[CR35] Wheeler M (2005). Reconstructing the cognitive world: the next step.

[CR36] Wheeler M (2008). Cognition in context: phenomenology, situated robotics and the frame problem. International Journal of Philosophical Studies.

[CR37] Wittgenstein L (1953). Philosophical investigations.

